# Accurate Prediction of Advanced Liver Fibrosis Using the Decision Tree Learning Algorithm in Chronic Hepatitis C Egyptian Patients

**DOI:** 10.1155/2016/2636390

**Published:** 2016-01-06

**Authors:** Somaya Hashem, Gamal Esmat, Wafaa Elakel, Shahira Habashy, Safaa Abdel Raouf, Samar Darweesh, Mohamad Soliman, Mohamed Elhefnawi, Mohamed El-Adawy, Mahmoud ElHefnawi

**Affiliations:** ^1^Informatics and Systems Department and Biomedical Informatics and Chemo Informatics Group, Engineering Research Division and Centre of Excellence for Advanced Sciences, National Research Centre, Giza, Egypt; ^2^Department of Endemic Medicine and Hepatology, Faculty of Medicine, Cairo University, Cairo, Egypt; ^3^Communications, Electronics and Computers Department, Faculty of Engineering, Helwan University, Cairo, Egypt; ^4^Hepatology & Endemic Medicine, Cairo University, Cairo, Egypt; ^5^Hepatology and Gastroenterology, Liver Unit, Cairo University, Cairo, Egypt; ^6^Communications and Computer Department, Faculty of Engineering, Modern University, Cairo, Egypt; ^7^Center for Informatics, Nile University, Giza, Egypt

## Abstract

*Background/Aim*. Respectively with the prevalence of chronic hepatitis C in the world, using noninvasive methods as an alternative method in staging chronic liver diseases for avoiding the drawbacks of biopsy is significantly increasing. The aim of this study is to combine the serum biomarkers and clinical information to develop a classification model that can predict advanced liver fibrosis. *Methods*. 39,567 patients with chronic hepatitis C were included and randomly divided into two separate sets. Liver fibrosis was assessed via METAVIR score; patients were categorized as mild to moderate (F0–F2) or advanced (F3-F4) fibrosis stages. Two models were developed using alternating decision tree algorithm. Model 1 uses six parameters, while model 2 uses four, which are similar to FIB-4 features except alpha-fetoprotein instead of alanine aminotransferase. Sensitivity and receiver operating characteristic curve were performed to evaluate the performance of the proposed models. *Results*. The best model achieved 86.2% negative predictive value and 0.78 ROC with 84.8% accuracy which is better than FIB-4. *Conclusions*. The risk of advanced liver fibrosis, due to chronic hepatitis C, could be predicted with high accuracy using decision tree learning algorithm that could be used to reduce the need to assess the liver biopsy.

## 1. Introduction

Chronic hepatitis C (CHC) is recognized as a major healthcare problem worldwide and as a common infection in Egypt, especially genotype 4 [1, 2]. The assessment of liver fibrosis in CHC is essential to monitor the prognosis of the disease, to establish the optimal timing for therapy and management strategies, and to predict the response to treatment [3]. Liver biopsy is considered as mandatory for the management of patients infected with the hepatitis C virus (HCV), particularly for staging of liver fibrosis degree. Some can consider it as a gold standard [4]. However, liver biopsy has potential risk due its limitations including its invasive nature, being costly, being susceptible to sampling error, and the histological assessment that may suffer from variability of results [5–7]. Therefore, in recent years the noninvasive methods have significantly increased in use as an alternative in staging chronic liver diseases for avoiding the drawbacks of biopsy.

Many noninvasive methods have been proposed to predict fibrosis and cirrhosis in patients with hepatitis C. Noninvasive methods should be safe, easy to perform, inexpensive, reproducible and give numerical and accurate results in real time [8]. There are two kinds of noninvasive methods: based on indexes derived from serum markers [9–11], such as FIB-4 score and the aspartate aminotransferase- (AST-) to-platelet ratio index (APRI) [12, 13], or based on imaging techniques, such as using Transient Elastography (TE), which uses ultrasound and vibratory waves for estimating the extent of liver fibrosis [14–16]. According to Parkes et al. [17], serum markers of liver fibrosis offer an attractive alternative to liver biopsy; they are less invasive than biopsy, with no risk of complications, eliminate sampling and observer variability, and can be performed repeatedly.

In recent years, machine-learning techniques such as classification trees and artificial neural networks (ANN) have been used as prediction, classification, and diagnosis tools [18–20]. Alternating decision tree (ADT) combines the simplicity of single decision tree with the effectiveness of boosting [21]. This study aims at combining the serum biomarkers and clinical information to develop a classification model that can differentiate between mild to moderate liver fibrosis and advanced fibrosis stages accurately and to evaluate the usefulness of using decision tree algorithms in prediction of advanced fibrosis.

## 2. Patients and Methods

### 2.1. Patients

This study was carried out on 39,567 patients that were enrolled in Egyptian National Committee for Control of Viral Hepatitis database in National Treatment Program of HCV patients in Egypt. They were 10741 females and 28826 males. The laboratory tests were performed at the same time of liver biopsy. The dataset of blood serum for the patients has been investigated and analyzed. The data contains reported clinical information such as age, gender, and body mass index (BMI), histological findings such as grade of fibrosis and the activity, and laboratory tests such as albumin, total bilirubin, indirect bilirubin, alanine aminotransferase (ALT), aspartate aminotransferase (AST), alpha-fetoprotein (AFP), postprandial glucose test (PC%), international normalized ratio (INR), quantity of HCV_RNA, white blood cells (WBC) count, hemoglobin (Hb), platelet count, creatinine, serology finding, glucose, postprandial glucose test (PC%), and platelet count.

All data were obtained on baseline, before starting antiviral therapy. Alcohol consumption was included in the questionnaire of the patients on baseline; most of the fields were missing or with denial of alcoholic consumption. Therefore and due to rare consumption of alcohol by Egyptian people, a specific history of alcohol consumption was not considered as covariant. The study was done under informed consent that was done by the National Committee for Control of Viral Hepatitis.

### 2.2. Liver Biopsy Histology

Liver histology is determined via METAVIR score [22] as assessed by local pathologists from Egypt. All patients underwent liver biopsy at baseline. Total histological activity index and fibrosis scores (F0–F4) were recorded. According to the METAVIR system, fibrosis was staged on a scale from F0 to F4, as follows: F0: no fibrosis; F1: portal fibrosis, without septa; F2: few septa; F3: many septa without cirrhosis; and F4: cirrhosis, respectively. F0 and F1 were considered as mild fibrosis and F2 as moderate, whereas F3-F4 were considered as advanced fibrosis [23].

### 2.3. Inclusion Criteria and Exclusion Criteria

Inclusion criteria were age ≥ 18 years and ≤60 years, positive HCV antibodies and detectable HCV RNA by PCR, positive liver biopsy for chronic hepatitis with F1 METAVIR score and elevated liver enzymes or F2/F3 METAVIR score, being naïve to treatment with PEG-IFN and RIB, hepatitis B surface antigen negativity, normal complete blood count, normal thyroid function, prothrombin concentration ≥ 60%, normal bilirubin, *α*-fetoprotein < 100 (ng/mL), and antinuclear antibody titer < 1/160.

Exclusion criteria were serious comorbid conditions such as severe arterial hypertension, heart failure, significant coronary heart disease, poorly controlled diabetes (hemoglobin A1C > 8.5%), chronic obstructive pulmonary disease, major uncontrolled depressive illness, solid transplant organ (renal, heart, or lung), untreated thyroid disease, history of previous anti-HCV therapy, body mass index (BMI) > 35 kg/m^2^, known human immunodeficiency virus (HIV) coinfection, hypersensitivity to one of the two drugs (PEG-IFN, RIB), and concomitant liver disease other than hepatitis C (chronic hepatitis B, autoimmune hepatitis, alcoholic liver disease, hemochromatosis, *α*-1 antitrypsin deficiency, and Wilson's disease).

### 2.4. Statistical Analysis, Feature Selection, and Classification

The data were statistically analyzed using the MedCalc software and Microsoft Excel, while Weka Software performed the decision tree learning. Data were reported as mean value ± standard deviation (SD). The relationship between variables and the presence of significant fibrosis has been assessed. The Kruskal-Wallis test has been used for continuous variables with nonnormal distribution. The chi-square test has been used for categorical variables. Pearson's correlation coefficients between fibrosis and each variable have been assessed.

We implemented several types of decision tree learning techniques such as classification and regression tree (CART) [24], C4.5 [25], reduced error-pruning tree (REP), and alternating decision tree [21]. We evaluated the performance of each of them on the datasets. The test set represents an external data set that was not used for training. The receiver operating curves (ROCs), sensitivities, specificities, predictive values, and accuracies were applied to evaluate the performance of each model or technique on both the training and test sets.

### 2.5. The Alternating Decision Tree

The alternating decision tree (ADT) is a classification and predictive learning machine method. Traditional boosting decision tree algorithms such as CART [24] and C4.5 [25] create complicated decision tree structures that are hard to interpret. ADTree merges a number of weak hypotheses to induce a boosted one. At the same time, classifiers of this type are easy to interpret [21]. An alternating decision tree, as any decision tree, consists of decision nodes and prediction nodes. Decision nodes specify a collection of attributes. The branches between the nodes convey the possible values that these attributes can have in the observed samples. Prediction nodes have a numeric score. In contrast, in ADT, prediction nodes exist as both root and leaves. An instance is classified in an ADT by following all paths for which all decision nodes are true and summing any prediction nodes that are traversed [26].

## 3. Results and Discussion

Liver fibrosis was staged (F0–F4) and required laboratory tests were performed. The distribution of fibrosis stages and the three strata among training and test sets were stated in Table 1. Patients were divided according to random uniform sampling into two separate sets. About two-thirds of the dataset were used for training (*n* = 22,690 patients) and the rest of data for test (*n* = 16,877 patients).  Table 2 states the characteristics of patients in training and test datasets and states the *P* value and Pearson correlation coefficients between each variable and fibrosis in training set. Data expressed as mean ± SD unless otherwise was stated. As recognized, the training and test sets were approximately close to each other. The correlation and *P* value results as shown in Table 2 identified age, body mass index (BMI), alpha-fetoprotein (AFP), aspartate aminotransferase (AST), platelets count, and albumin as independent predictors of fibrosis, with highest statistically significant relationship (*P* value < 0.0001) and accepted correlation (|*r* | >0.1) with fibrosis. Therefore, these variables were used in model 1.

**Figure 1:**
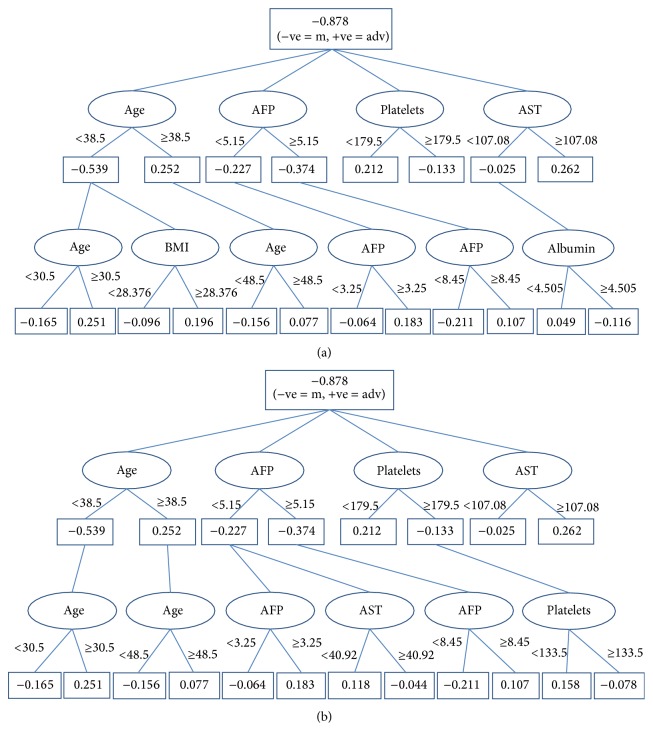
Decision tree diagrams. (a) Model 1. (b) Model 2. Advanced fibrosis is considered as the positive, referred to by symbol (adv), while moderate or mild fibrosis is considered as negative and referred to by symbol (m). The liver fibrosis of the patient is scored by summing all of the prediction nodes through which it passes.

In model 1, alternating decision tree was learned for the training data set considering the six variables (which are statistically significant relationship (*P* value < 0.0001) and accepted correlation coefficients (|*r* | >0.1) with fibrosis): age, body mass index (BMI), alpha-fetoprotein (AFP), aspartate aminotransferase (AST), platelet count, and albumin.  Figure 1(a) shows the decision tree diagram of model 1. In Figure 1(a), advanced fibrosis is considered as positive, referred to by symbol (adv), while moderate or mild fibrosis is considered as negative, referred to by symbol (m). The liver fibrosis of the patient is scored by summing all of the prediction nodes through which it passes. If the result is more than or equal to zero, then the patient is high risked to have advanced fibrosis and vise a versa.

The four variables age, AFP, platelet count, and AST have the least *P* value and the most correlated coefficients according to our previous work on the subject [11]; therefore in the study we investigate if we can exclude BMI and albumin from the effective prediction features. In model 2, alternating decision tree was learned for the training data set considering the four variables: age, alpha-fetoprotein (AFP), aspartate aminotransferase (AST), and platelet count. These features were similar to FIB-4 features except AFP instead of ALT.  Figure 1(b) shows the decision tree diagram of model 2.

**Figure 2:**
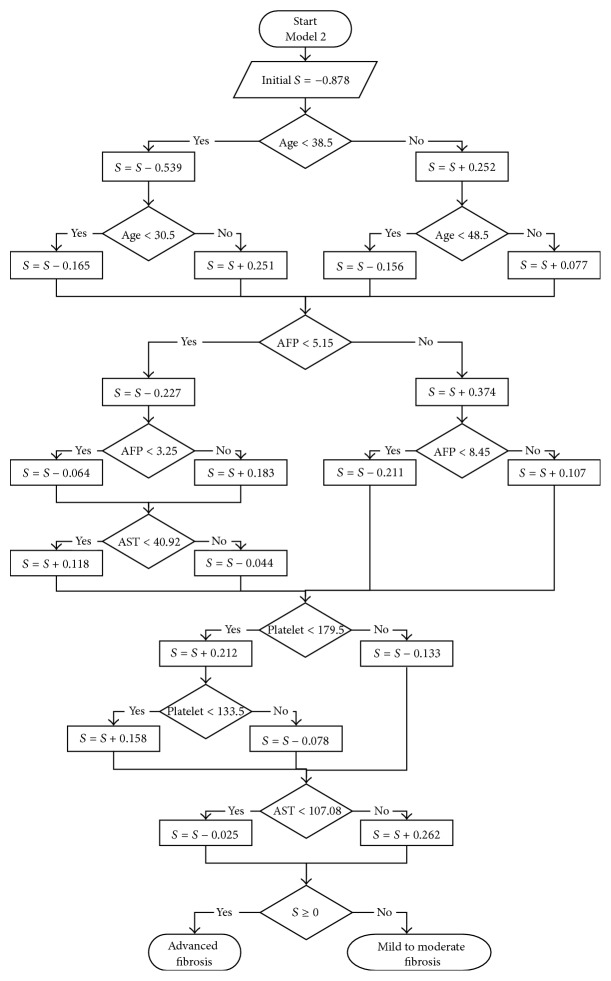
Flowchart of model 2. *S* represents the fibrosis score of the patient. If final *S* ≥ 0, then the patient has an advanced fibrosis and vice versa.

For more explanation, Figure 2 represents the flow chart of model 2. The liver fibrosis of the patient is scored by summing all of the prediction nodes through which it passes. Each positive value of prediction nodes boosts the probability of having an advanced fibrosis or decreases it by negative value. If the result was positive (≥0), then it was predicted that the patient has an advanced fibrosis. If the result was negative (<0), then it was predicted that the patient does not have an advanced fibrosis. For example, a 45-year-old patient with AFP of 9.7 U/L, AST of 394 U/L, and platelet a count of 139 × 10^9^/L would have (score = −0.878 + 0.252 − 0.156 + 0.374 + 0.107 + 0.212 − 0.078 + 0.262 = 0.095). The final score of 0.095 is positive; as the criteria value is zero in the ADT techniques, so the patient can be classified to have an advanced liver fibrosis, conforming the fibrosis biopsy result of that patient, which was F3.

**Figure 3:**
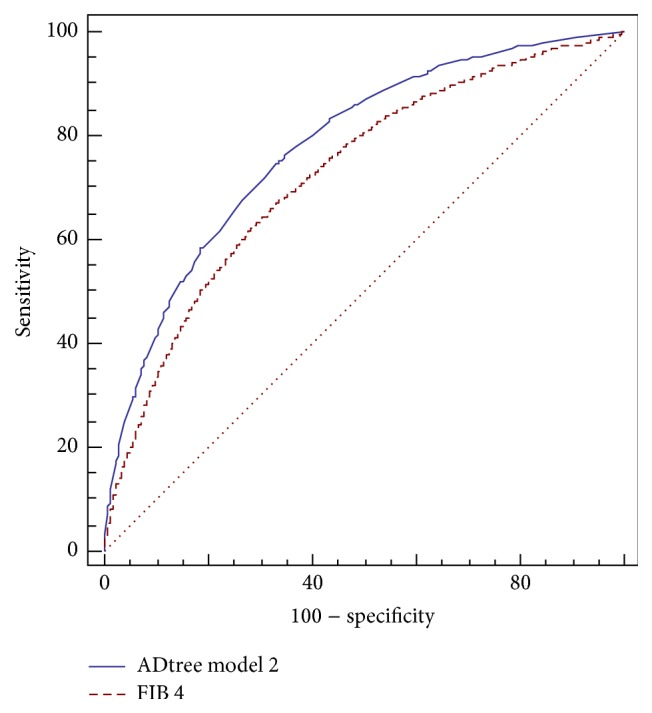
Comparison between the ROC curves of proposed ADTree Model 2 and FIB 4. It shows an improved AUROC for model 2.

Table 3 states the accuracy, ROC analysis, sensitivity, specificity, and positive and negative predictive values of model 1 and model 2 for predicting advanced fibrosis in training set and shows a comparison between the results of these models and FIB-4 algorithm on the test set. Model 2 achieved highest accuracy of 85.7% in training set and 84.8% in test set. Moreover, it shows the highest negative predictive value NPV 87.3% in training set and 86.2% in test set.  Figure 3 shows comparison between the ROC curves of proposed ADT model 2 and FIB 4. The areas under the ROC curves whether using model one or two are closer to each other (0.78), and better than the area under the ROC curve of FIB-4 (0.73). When we applied alternating decision tree algorithm (ADT) on cohort data with the six effective variables using cross validation with 10-fold, it achieved 0.78 ROC and 85.3% accuracy, which is very close to the results of using training and test sets separately. The low sensitivity of the models can be attributed to the zero cut-off frequency, which had been selected by ADT algorithm. The ADT algorithm trained at cut-off point zero. We can choose any other cut-off point from the ROC curve to increase the sensitivity but this will be at the expense of the accuracy and the ROC values.

As shown in Figure 3, the comparison between the ROC curves of proposed ADT model 2 and FIB 4 shows the preference of the proposed model 2, where there is a difference of 5% in the area under the ROC curve and of 2.5% in the accuracy in the interest of model 2.

## 4. Conclusion

In this study, we conclude that we can accurately predict advanced fibrosis stage for chronic HCV patients using learning decision trees with high accuracy. The most important features in predicting the advanced fibrosis were age, AFP, AST, and platelet count as they have the least *P* value and the most correlated coefficients as shown in the results of the proposed model. The best model achieved 86.2% NPV, 0.78 ROC, and 84.8% accuracy on the test set, better than classical FIB-4 method. The use of alpha-fetoprotein AFP as a feature of predicting advanced fibrosis in addition to using ADT improves the results compared to those of FIB-4 algorithm which uses ALT instead. The proposed model could be used as an acceptable, safe, and low cost alternate for predicting advanced fibrosis rather than relatively risky alternative tools (such as the liver biopsy) in chronic Egyptian hepatitis C virus patients.

## Figures and Tables

**Table 1:** Fibrosis records and strata in the data sets.

Fibrosis stage	Training dataset (*n* = 22690)	Test dataset (*n* = 16877)
0	34	42
1	11808	8337
2	7507	5821
3	3170	2513
4	171	164
Total	22690	16877
Fibrosis strata		
Mild to moderate (0–2)	19349	14200
Advanced (3-4)	3341	2677

**Table 2:** Characteristics of variables in coherent dataset.

Characteristics	Training dataset 22690	Validation dataset 16877	Pearson correlation coefficients	*P* value
Age (yrs)	40 ± 11	40 ± 10	0.26	<0.0001
Gender				
Female	6186 (27.3%)	4555 (26.9%)	−0.03	0.008
Male	16504 (72.7%)	12322 (73.1%)		
BMI	26.70 ± 3.79	26.79 ± 3.84	0.10	<0.0001
AFP (U/L)	7.26 ± 26.61	7.69 ± 28.49	0.10	<0.0001
ALP (U/L)	105.41 ± 65.17	105.41 ± 65.17	0.02	0.008
AST (U/L)	57.27 ± 33.73	56.78 ± 34.61	0.12	<0.0001
ALT (U/L)	61.84 ± 36.89	61.84 ± 38.19	0.06	0.008
Platelet count (*∗*10^9^/L)	212.48 ± 60.64	211.55 ± 60.86	−0.18	<0.0001
Albumin (g/dL)	4.39 ± 0.42	4.40 ± 0.42	−0.14	<0.0001
Indirect bilirubin (mg/dL)	0.57 ± 1.77	0.60 ± 2.24	−0.00	0.088
Total bilirubin (mg/dL)	0.76 ± 0.28	0.76 ± 0.28	0.05	<0.0001
Glucose (mg/dL)	96.57 ± 19.41	96.69 ± 20.71	0.08	<0.0001
Hemoglobin (Hb)	14.03 ± 1.47	14.03 ± 1.62	−0.00	0.0005
WBC (10^9^/L)	6.44 ± 1.90	6.44 ± 1.94	−0.02	0.0001

**Table 3:** Accuracy and Roc analysis of model 1, model 2, and FIB-4 for predicting advanced fibrosis with criteria value zero.

Model	Sensitivity %	Specificity %	PPV %	NPV %	ROC	Accuracy %
Model 1^*∗*^	15.1	97.9	55.2	87	0.78	85.7
Model 2^*∗*^	17.5	97.5	54.8	87.3	0.78	85.7
Model 1^*∗∗*^	14.4	97.9	56.6	85.9	0.78	84.7
Model 2^*∗∗*^	17.4	97.5	56.6	86.2	0.78	84.8
FIB-4^*∗∗*^	17.9	95.51	42.8	86.1	0.73	83.2

PPV: positive predictive value; NPP: negative predictive value; ROC: receiver operating characteristic curve.

^*∗*^Applying the model on the training set.

^*∗∗*^Applying the model on the test set.
